# Supplementary figures and images for: MTPrompt-PTM: A Multi-Task Method for Post-Translational Modification Prediction Using Prompt Tuning on a Structure-Aware Protein Language Model
Source: Biomolecules. 2025 Jun 9;15(6):843. doi: 10.3390/biom15060843 (PMC12190693; doi:10.3390/biom15060843)

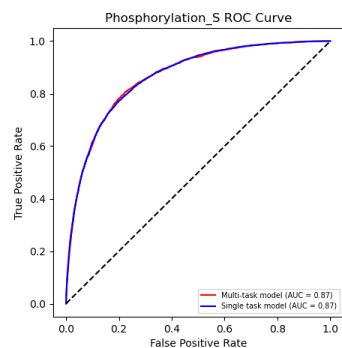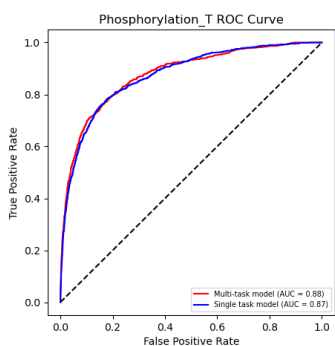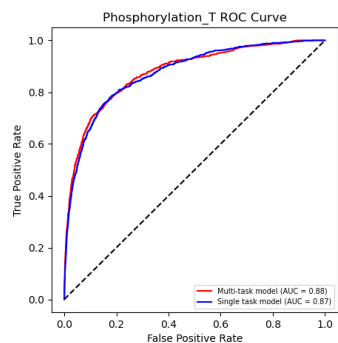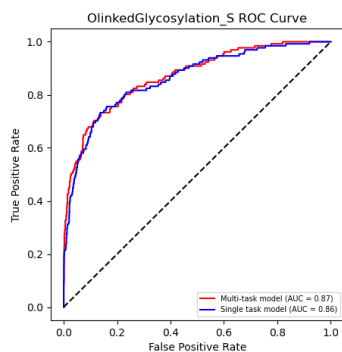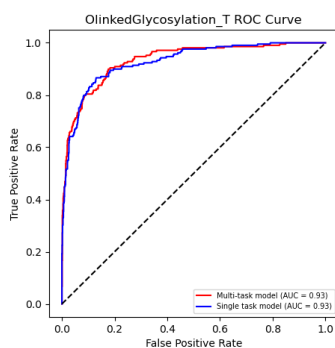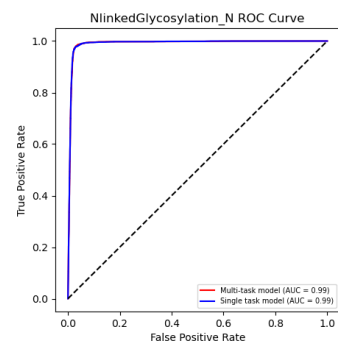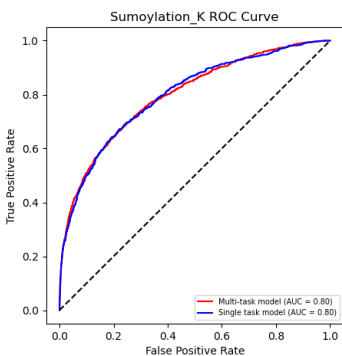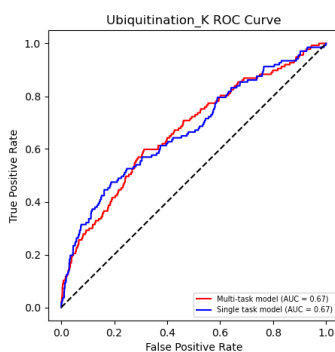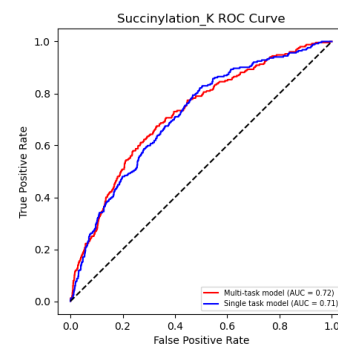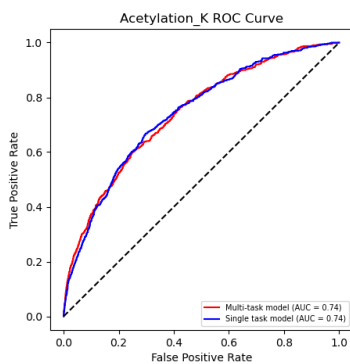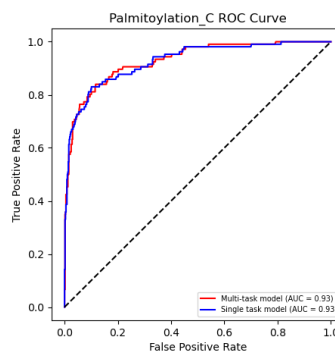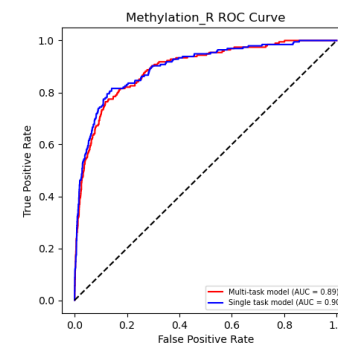

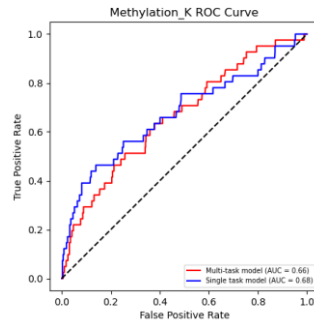

**Figure S1.** Performance comparison of AUROC between multi-task and single-task on 13 PTM types.

Supplement: Supplementary file 1 [file biomolecules-15-00843-s001.zip › biomolecules-3651358-supplementary/Supplementary/Supplementary-0430.pdf]
